# The role of empathy between peers in upper secondary students’ study engagement and burnout

**DOI:** 10.3389/fpsyg.2022.978546

**Published:** 2022-09-30

**Authors:** Lotta Tikkanen, Henrika Anttila, Kirsi Pyhältö, Tiina Soini, Janne Pietarinen

**Affiliations:** ^1^School of Applied Educational Science and Teacher Education, University of Eastern Finland, Joensuu, Finland; ^2^Faculty of Educational Sciences, University of Helsinki, Helsinki, Finland; ^3^Centre for Higher and Adult Education, Faculty of Education, Stellenbosch University, Stellenbosch, South Africa; ^4^Faculty of Education and Culture, Tampere University, Tampere, Finland; ^5^Philosophical Faculty, University of Eastern Finland, Joensuu, Finland

**Keywords:** adolescents, affective empathy, cognitive empathy, empathy, study burnout, study engagement, structural equation modeling

## Abstract

Having the ability to understand emotionally how other people feel and see things is an essential fabric for building and sustaining functional interpersonal relationships. Without such an ability, social interaction crumbles, engagement fails, and learning is eroded. Yet, empirical evidence on the relationship between study burnout and study engagement, and empathy between upper secondary school students is limited. We are tackling the challenge by exploring the association between empathy between peers and study engagement and study burnout among upper secondary school students. Two hundred and eighty upper secondary education students took part in our cross-sectional study. Structural equation modeling was used to analyze the association between empathy (i.e., cognitive and affective empathy), and study burnout and study engagement. The results showed that cognitive empathy contributed to affective empathy, which was further related to increased levels of study engagement, and decreased levels of cynicism, and sense of inadequacy. The role of cognitive empathy seemed to be more complicated: while cognitive empathy contributed directly to increased levels of cynicism, and inadequacy and decrease in study engagement, the indirect effects of cognitive empathy (through affective empathy) on cynicism and inadequacy were negative, and positive on study engagement. Neither of the empathy dimensions explained students’ emotional exhaustion. The results indicate that merely teaching students to recognize and identify their peers’ emotions is not sufficient to enhance study wellbeing, but they need to learn to share emotions and to tune into each other’s emotions.

## Introduction

The diminishing levels of study wellbeing have become a growing concern among educational practitioners, policymakers, and researchers worldwide (e.g., Walburg, 2014; Yang and Chen, 2016; Salmela-Aro et al., 2021; Vinter et al., 2021). In Finland, it has been suggested that up to 21% of upper secondary education students have suffered from study burnout, whereas 33% reported experiencing positive study engagement (Finnish Institute for Health and Welfare, 2021). The challenge has been further added by the lock downs caused by the COVID-19 pandemic. Increased study burnout and reduced study engagement have severe consequences for both the individual and the society, including learning loss, increased risk of depression, and dropping out from school (e.g., Salmela-Aro et al., 2009b; Bask and Salmela-Aro, 2013; Symonds et al., 2016). Accordingly, efficient means to buffer study burnout and enhance study engagement are called after.

Peer relationships have been shown to play a key role in the development of study wellbeing (e.g., Ryan, 2001; Rubin et al., 2008; Mendoza and King, 2020). Respectively, problems in this domain can dilute efforts to promote students’ study wellbeing made by teachers. Functional peer interaction provides the ability to recognize and identify the peer’s emotions and tune into each other’s emotional experiences; in other words, the empathy skills. Empathy skills are the core of receiving and providing well fitted social support that prevents study burnout and enhances study engagement among upper secondary school students, we believe. However, the effects of empathy on burnout and engagement so far have been explored primarily among healthcare professionals, social workers, teachers, and students within related fields (e.g., Wagaman et al., 2015; MacArthur et al., 2021; Wing et al., 2021). Accordingly, there is insufficient understanding on whether and how upper secondary education students’ empathy skills are related to their study burnout and study engagement. We have contributed to filling this gap in the research by exploring the association between upper secondary education students’ cognitive and affective empathy, and study burnout and study engagement.

### Study wellbeing

Study wellbeing is a multidimensional construct referring to positive mental states, such as satisfaction, self-efficacy, and/or study engagement, combined with the absence of negative ones, such as study burnout or strain related to the studying, that together contribute to successful studying (Korhonen et al., 2014; Widlund et al., 2018). Study wellbeing is constructed through interactions between the students and their learning environment (Salmela-Aro and Upadyaya, 2014). In this study, we focused on upper secondary education students’ study wellbeing in terms of study engagement and study burnout.

*Study engagement* is characterized by vigor, dedication, and absorption (Schaufeli et al., 2002; Salmela-Aro and Upadyaya, 2012), and it has been suggested that it is a symbol of an optimal learning experience. The positive association between students’ study engagement, life satisfaction and success in educational transitions has been detected in previous studies (see e.g., Salmela-Aro and Tuominen-Soini, 2010; Lewis et al., 2011). In turn, *study burnout* refers to a negative study experience resulting from prolonged study-related stress, characterized by three symptoms: *emotional exhaustion*, *cynicism*, and *sense of inadequacy* (Salmela-Aro et al., 2009a). *Emotional exhaustion* refers to chronic fatigue and lack of emotional energy, *cynicism* means alienation from the studying and perceiving the studying as meaningless, and *sense of inadequacy* is characterized by reduced sense of accomplishment in studying (Salmela-Aro et al., 2009a; Salmela-Aro and Upadyaya, 2014). There is evidence that study burnout is related to other negative mental states, such as depression, and dropping out (e.g., Salmela-Aro et al., 2009b; Bask and Salmela-Aro, 2013; Symonds et al., 2016). Both development of study burnout and study engagement are socially embedded (e.g., Kiuru et al., 2008; Mendoza and King, 2020). This means that the quality and quantity of interactions in school with teachers and peers can either decrease or increase students’ study wellbeing.

Peers play a significant role in the development of study engagement and burnout among students (see Ryan, 2001; Rubin et al., 2008; Mendoza and King, 2020). The quality of peer interaction and the effect it has on study wellbeing are dependent on the students’ socio-emotional skills. However, prior studies focused heavily on the effect of identification and regulation of one’s own emotions on study burnout and study engagement (e.g., Usán Supervía et al., 2019; Salmela-Aro and Upadyaya, 2020; Vinter et al., 2021). Empathy skills, such as abilities to recognize peers’ emotions and abilities in perspective taking and tuning into each other’s emotions, which can be assumed to be crucial in functional peer interaction, and further, in the optimal development of study wellbeing, have rarely been the focus of those studies.

### Empathy between peers

Empathy refers to an emotional response to the affective state or situation of another person (e.g., Feshbach and Roe, 1968; Eisenberg et al., 1991). Empathy has two dimensions: *cognitive empathy*, referring to ability to recognize and understand another’s feelings, while *affective empathy* entails one’s ability to share emotions or tune into another’s emotional experiences (Eisenberg, 2004). It has been suggested that the cognitive and affective dimensions of empathy are distinct but related constructs playing different but complementary roles in empathy (see Jolliffe and Farrington, 2006). The cognitive dimension includes the ability to understand how peers are thinking and feeling, but not necessarily sharing the feelings of another or resonating with those feelings (i.e., affective dimension). That the cognitive dimension might be a prerequisite for affective dimension has been proposed (see Feshbach and Roe, 1968; Lamm et al., 2010).

Previous studies have shown that empathy is positively associated with psychological wellbeing, positive behavior, such as prosocial behavior and problem solving, and negatively with antisocial behaviors such as aggressive behavior and engagement in conflicts (de Wied et al., 2006; Van Lissa et al., 2016; Laghi et al., 2018; van der Graaff et al., 2018; Vinayak and Judge, 2018). Low levels of empathy have been shown to be related to conflicts, aggression and bullying, respectively (Euler et al., 2017). Although the assets of well-developed empathy skills have been highlighted in the previous literature, it has also been suggested that it has downsides. For example, an association between high levels of empathy and internalizing problems, such as depression, has been detected (Calandri et al., 2019, 2021). The cognitive and affective dimension seem to be differentially associated with behavioral outcomes. The cognitive dimension has been shown to be positively related to indirect forms of aggression, while affective dimension has been associated with a decrease in relational and overt aggression (Batanova and Loukas, 2011, 2012). Moreover, without sufficient skills to overcome negative mental states, high levels of affective empathy may lead to co-rumination and increased risks of experiencing burnout symptoms *via* crossover or contagion (see Schwartz-Mette and Rose, 2012; Boren, 2013).

It has been shown that learning environments provided by a school play a role in the development of empathy and hence potentially in the development of study wellbeing: for example, receiving adequate support and acceptance from the teachers and peers can help students to learn to take others’ perspectives (Batanova and Loukas, 2012). Farina et al. (2020) detected a positive association between students’ affective empathy and exhaustion and showed that empathy as a whole contributed to higher levels of study burnout. High levels of empathy may contribute to the crossover of study burnout and study engagement by increasing the likelihood of emotional contagion (see Hatfield et al., 1992; Mendoza and King, 2020). This means that the students with high levels of empathy can be assumed to be more likely to “catch” their peers’ study-related emotional states – both positive and negative – that can further contribute to their own study wellbeing, than students with low empathy levels. Also gendered differences in empathy have been detected. Girls typically report higher levels of empathy than boys (e.g., Jolliffe and Farrington, 2006; Mestre et al., 2009; Calandri et al., 2021). Moreover, individual variations in students’ empathy levels are likely to occur. For example, adolescents are likely to show more empathy for their peers than for their teachers or parents due to their experience of similar life conditions. However, to our knowledge, there are no measures of students’ empathy specifically designed for measuring students’ empathy toward their peers in educational settings. The empirical evidence of the role of empathy dimensions in study burnout symptoms and study engagement is limited. Hence, our study contributes to filling this gap by exploring the association between students’ affective and cognitive empathy toward their peers, and study burnout symptoms and study engagement.

### Aim of the study

The aim of this study was to advance the understanding of the function of students’ empathy in educational settings by exploring the relationship between empathy and study wellbeing among upper secondary education students. First, we introduced an instrument for measuring students’ empathy toward peers in educational settings and explored whether gender explained variation in empathy dimension (see e.g., Jolliffe and Farrington, 2006; Mestre et al., 2009; Calandri et al., 2021). Second, as the previous studies have not covered the effects of empathy skills on students’ study well-being sufficiently, we explored the interrelations between cognitive and affective empathy and students’ experiences of study burnout symptoms and study engagement (see Farina et al., 2020). Last, based on previous literature (see Feshbach and Roe, 1968; Lamm et al., 2010), we expected the cognitive empathy to be a prerequisite for affective empathy, and hence, explored the indirect effects of cognitive empathy (mediated by affective dimension) on study well-being. The following hypotheses were tested:

**H1:** Students’ empathy skills comprise two components: (1) cognitive empathy and (2) affective empathy.

**H2:** Gender explains the variation in cognitive empathy (CE) and affective empathy (AE) (Jolliffe and Farrington, 2006; Calandri et al., 2021).

**H3:** The cognitive dimension of empathy (CE) explains the variation in the affective dimension of empathy (AE) (see Feshbach and Roe, 1968; Lamm et al., 2010).

**H4:** Both dimensions of empathy are directly related to study engagement (ENG) and study burnout symptoms, i.e., exhaustion (EXH), cynicism (CYN), and inadequacy (INAD) (Farina et al., 2020).

**H5:** The affective dimension of empathy mediates the association between cognitive empathy and study engagement (ENG), and study burnout symptoms (EXH, CYN, and INAD).

## Materials and methods

### Research context

Finnish children typically start their school career with pre-primary education at the age of six. At the age of seven, they start their 9 years of comprehensive schooling, consisting of primary (grades 1–6) and lower secondary schools (grades 7–9). After comprehensive school, at about the age of 16, the adolescents apply for entry into upper secondary education, which is either senior high school (academic track) or vocational schools, or a combination of these. Some students opt to attend a voluntary tenth grade. In the year of 2020, 54% of the adolescents who had completed the comprehensive school entered the upper secondary academic track, and 39% entered the vocational track (Statistics Finland, 2020).

### Participants

The participants in the study were 280 students in upper secondary education. They were about 17 years old. Seventy-eight percent of them took the academic track (*n* = 217) and 21% took the vocational track (*n* = 60). Most of the participants were girls (69%, *n* = 192), while minority were boys (28%, *n* = 79). Of the participants, 3% (*n* = 9), disclosed “other” as their gender or did not want to specify it. Girls, and those on the academic track were overrepresented in the sample. The participants were from all over Finland, and both high (61%) and low (39%) socioeconomic neighborhoods in rural and urban areas were represented.

### Data

The data collection was part of a larger, longitudinal research project. The participants had been involved in the study for 4 years, i.e., since 2017, when they were in the seventh grade. The data used in this study were collected between May and June 2021. The participants who had given their contact information (*N* = 761) and the permission to be contacted regarding the follow-up in the third stage of the data collection (in 2019) were sent a link to the online survey *via* SMS and e-mail. The data were collected in the second year of their studying in upper secondary education, and they were about 17 years old. All the participants were informed about the study, and the participation was voluntary. They also gave their informed, written consent to participate in the study. Four gift cards (€100/each to verkkokauppa.com) were drawn as an incentive to participate in the study.

In Finland, ethical review is required when research involves intervention in the physical integrity of research participants, deviates from the principle of informed consent, involves participants under the age of 15 being studied without parental consent, exposes participants to exceptionally strong stimuli, risks causing long-term mental harm beyond that encountered in normal life, or signifies a security risk to subjects (Finnish National Board on Research Integrity, 2019, p. 19). None of these conditions were encountered in this study, and therefore no ethics review was required.

### Measurement

In this study, we used the following scales: (1) empathy toward peers, consisting of two factors: *cognitive empathy* (five items) and *affective empathy* (four items), and (2) *study engagement* (nine items) (Salmela-Aro and Upadyaya, 2012), and (3) study burnout consisting of three factors: *emotional exhaustion* (three items), *cynicism* (two items), and *sense of inadequacy* (two items) (Salmela-Aro et al., 2009a). In addition, background variable, gender (female/male) was used. The *empathy toward peers*-scale was developed for the study by the authors. The cognitive empathy scale was modified from The Cognitive, Affective, and Somatic Empathy Scales (CASES) (Raine and Chen, 2018), and the affective empathy scale was inspired by Questionnaire measure of empathic tendency (Mehrabian and Epstein, 1972) and Multidimensional Emotional Empathy Scale (MDEES) (Alloway et al., 2016). We developed six items to measure cognitive empathy and six items for emotional empathy. The items were formulated to suit the educational context, and the items covered both positive and negative social, study-related emotions. Before data collection, the *empathy toward peers*-scale was tested and further developed based on a pilot study. In the pilot study, 22 upper secondary education students completed the survey and commented on the items. Cronbach’s alphas were calculated for the factors (i.e., cognitive empathy and affective empathy) and based on these results, some of the items were removed.

### Analyses

The descriptive statistics of the study variables, the correlations between them, and gendered differences in empathy dimension were calculated with IBM SPSS Statistics 28.0. Other analyses were conducted using Mplus version 8.6.

First, the factorial structure of each scale (cognitive empathy, affective empathy, study engagement, and study burnout) was tested separately using confirmatory factor analyses. The measurement models were estimated using an MLR procedure which produces maximum likelihood estimates with standard errors and Chi-square test statistics that are robust to non-normality (Muthén and Muthén, 1998–2017), and the full information maximum likelihood method using all the information that is available in the data (Schafer and Graham, 2002). The goodness-of-fit of the estimated standardized models was assessed using a Chi-square test, Comparative Fit Index (CFI), Tucker–Lewin Index (TLI), Root Mean Square Error of Approximation (RMSEA), and Standardized Root Mean Square Error of Approximation (SRMR). Correlations between some of the residuals were added to the measurement models when they significantly improved the model and were substantively meaningful (Byrne, 2012). Item reliability was explored by estimating the reliability coefficients (R-squared; Supplementary Appendix 1) and the structural validity by estimating the standardized factor loadings (Supplementary Appendix 1; Hair et al., 2014). The internal consistency of the scales was examined by the factor determinacies and Cronbach alphas. Discriminant validity was explored by comparing the square root of the average variance extracted (AVE) values for each construct with the correlations between the different constructs (Fornell and Larcker, 1981) and by analyzing with Wald tests whether the correlations between the latent variables differed from one. Furthermore, multi-collinearity of the scales was examined using VIF (variance inflation factor) scores.

Second, the structural equation modeling (SEM) was used to determine the extent to which the hypothesized model was consistent with the data (Muthén and Muthén, 1998–2017; Byrne, 2012). The MLR procedure and the full information maximum likelihood were used (Schafer and Graham, 2002). The goodness-of-fit of the estimated standardized models was assessed using a Chi-square test, CFI, TLI, RMSEA, and SRMR. Gender was included in the model as an observed binary predictor variable. Other variables were included as latent variables. Independent samples *t*-test was used to further investigate the gendered differences in cognitive and affective empathy.

## Results

The CFA results (see Supplementary Appendix 1) showed that the measurement models of students’ cognitive empathy [χ^2^(5) = 10.22, *p* = 0.07, CFI/TLI = 0.994/0.988, RMSEA = 0.061 (90% CI: 0.00–0.12), SRMR = 0.015] and affective empathy [χ^2^(1) = 4.35, *p* < 0.05, CFI/TLI = 0.995/0.972, RMSEA = 0.109 (90% CI: 0.02–0.22), SRMR = 0.009) toward their peers fitted the data (H1). *Cognitive empathy* reflected students’ skills in recognizing and understanding their peers’ emotional experience and perspective taking, while *affective empathy* focused on students’ skills in tuning into their peers’ emotions and reacting to them. The construct validity of the scales was considered sufficient from the perspectives of convergent construct validity and discriminant construct validity. The standardized factor loadings were adequate between observed variables and latent variables (≥0.50) (see Supplementary Appendix 1). The internal consistencies of the empathy scales were considered acceptable according to their Cronbach Alphas (Table 1), CR (>0.70) and AVE (>0.50) values, and factor determinacies (see Supplementary Appendix 1). In addition, discriminant validity between the empathy scales and the study burnout factors and study engagement scale was supported, with the square root of the AVE of each construct being higher than the correlation between the different constructs (Fornell and Larcker, 1981). The Wald tests also showed that the correlations between the latent factors differed significantly from one (*p* < 0.001) supporting sufficient discriminant validity between the scales. There were no signs of multi-collinearity either, as the VIF values of the empathy scales were below the threshold of 3.3 (Kock and Lynn, 2012).

**TABLE 1:** The descriptive statistics of the study variables and correlations between them.

	1. Cognitive empathy	2. Affective empathy	3. Study engagement	4. Exhaustion	5. Cynicism	6. Inadequacy
1. Cognitive empathy						
2. Affective empathy	0.670**					
3. Study engagement	0.215**	0.347**				
4. Exhaustion	0.052	−0.024	−0.297**			
5. Cynicism	−0.103	−0.275**	−0.553**	0.454**		
6. Inadequacy	0.044	−0.080	−0.392**	0.739**	0.626**	
No. of items	5	4	9	3	2	2
Cronbach’s α	0.895	0.882	0.949	0.826	0.922	0.848
Mean	5.65	5.52	3.92	4.19	3.06	3.95
SD	1.02	1.12	1.39	1.61	1.80	1.82
Min/max	1.20/7	1/7	1/7	1/7	1/7	1/7

**p < 0.001.

On average, the students reported both high levels of cognitive empathy and affective empathy toward their peers (see Table 1). They reported increased levels of exhaustion and inadequacy, and moderate levels of cynicism. At the same time, the students reported experiencing elevated levels of study engagement. The study burnout symptoms correlated positively with each other and negatively with engagement. The cognitive and affective empathy were positively related to study engagement, and affective empathy was negatively related to cynicism.

**FIGURE 1:**
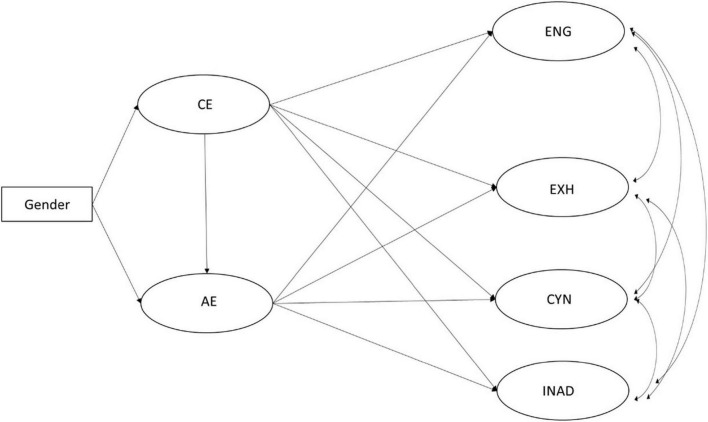
Hypothesized model of the interrelations between the dimensions of empathy and study wellbeing.

**FIGURE 2:**
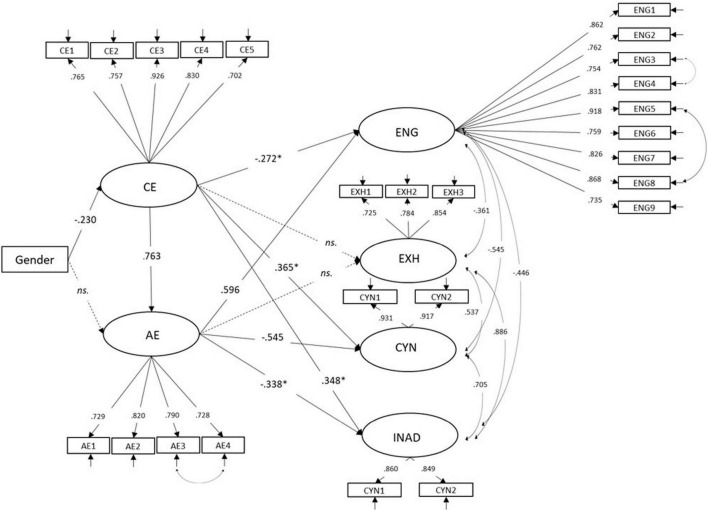
The structural equation model of the relations between latent variables of cognitive empathy (CE), affective empathy (AE), study engagement (ENG), emotional exhaustion (EXH), cynicism (CYN), and inadequacy (INAD). Standardized model. *ns.*, indicates non-significant paths, *p < 0.05, all other parameters are significant at the *p* < 0.001 level.

The hypothesized model (Figure 1) was consistent with the data [χ^2^(95) = 458.37, *p* < 0.001, RMSEA = 0.048 (90% CI: 0.040–0.056), CFI = 0.959, TLI = 0.952, SRMR = 0.062]. The regression coefficients are shown in Figure 2. As was hypothesized (H2), gender had an effect on cognitive empathy, which meant that girls scored higher in the cognitive dimension of empathy than boys. However, gender did not have direct effect on affective empathy. Further investigation on indirect effects (see Table 2) showed that there was a significant indirect path from gender to affective empathy. This indicates that, in the model, the girls who scored higher in cognitive empathy also scored higher in affective dimension, but gender did not contribute directly to variation in affective empathy. The differences between the means of cognitive empathy (girls: *M* = 5.82, SD = 0.95; boys: *M* = 5.35, SD = 1.04) and affective empathy (girls: *M* = 5.70, SD = 1.02; boys: *M* = 5.19, SD = 1.15) were significant [cognitive empathy: *t*(269) = −0.3.55, *p* < 0.001; affective empathy: *t*(269) = −3.57, *p* < 0.001]. The cognitive empathy contributed to higher levels in affective empathy (H3). This means that the students who recognized and identified their peers’ emotions skillfully, also tended to react and tune into emotional states of another person more easily. Some of the hypothesized direct effects of the empathy dimensions on study wellbeing were also detected (H4): The cognitive empathy was related to increased levels of cynicism and a sense of inadequacy. Cognitive empathy also had a direct negative effect on study engagement. Affective empathy was related to diminished levels of cynicism and sense of inadequacy, and increased levels of study engagement. However, neither dimension of empathy was related to exhaustion. These direct paths from empathy dimensions to study wellbeing suggest that the students’ skills in recognizing and identifying their peers’ emotional states might contribute to diminished levels of study engagement and increased levels of cynicism and inadequacy, while the students’ skills in reacting and tuning into others’ emotional states might protect them from cynicism and inadequacy and increase the levels of study engagement.

**TABLE 2:** Standardized indirect estimates, confidence intervals, and *p*–values.

	Effect	Lower 2.5%	Upper 2.5%	SE	*p*
**Effects of cognitive empathy on study engagement**					
Total	0.183	0.060	0.307	0.063	0.004
Direct	−0.272	−0.505	−0.038	0.119	0.023
Indirect (through affective empathy)	0.455	0.247	0.663	0.106	0.000
**Effects of cognitive empathy on exhaustion**					
Total	0.085	−0.053	0.222	0.070	0.227
Direct	0.223	−0.024	0.470	0.126	0.077
Indirect (through affective empathy)	−0.139	−0.346	0.069	0.106	0.191
**Effects of cognitive empathy on cynicism**					
Total	−0.051	−0.179	0.078	0.066	0.441
Direct	0.365	0.124	0.605	0.123	0.003
Indirect (through affective empathy)	−0.415	−0.629	−0.202	0.109	0.000
**Effects of cognitive empathy on inadequacy**					
Total	0.091	−0.038	0.219	0.066	0.166
Direct	0.348	0.083	0.614	0.135	0.010
Indirect (through affective empathy)	−0.258	−0.490	−0.025	0.118	0.030
**Effects of gender on affective empathy**					
Total	−0.225	−0.360	−0.090	0.069	0.001
Direct	−0.049	−0.173	0.074	0.063	0.432
Indirect (through cognitive empathy)	−0.175	−0.278	−0.073	0.052	0.001

Further investigation of the indirect effects (see Table 2) showed that the affective dimension mediated the association between cognitive empathy and study wellbeing (H5): significant negative, indirect effects from cognitive empathy to inadequacy and cynicism were detected. Furthermore, an indirect positive effect was detected from cognitive empathy to study engagement. In other words, the affective empathy seemed to mediate the effect of cognitive empathy on cynicism, sense of inadequacy, and study engagement (H5). These indirect paths indicate that when students’ skills in recognizing their peers’ emotional states contributed to higher levels of affective empathy, they also had a positive effect on study wellbeing in terms of diminished cynicism and inadequacy, and increased study engagement.

## Discussion

### Findings in the light of previous literature

The aim of this study was to advance the understanding of the function of students’ empathy in educational settings by exploring the relationship between empathy and study wellbeing among students. First, we introduced an instrument for measuring students’ empathy toward peers in educational settings. Second, we explored the interrelations between cognitive and affective empathy, and students’ experiences of study burnout symptoms and study engagement. The results showed that the students had well-developed empathy skills between themselves and their peers in terms of both the cognitive dimension and affective dimension; that is, they skillfully recognized if their peers’ emotions in different situations and reacted easily to their peers’ emotions. Being able to empathize with others allows us to relate with each other. It makes our thoughts, emotions, and behaviors more alike, creating social glue and hence allowing students to build and sustain relationships with their peers. Based on our findings it seems that cognitive and effective empathy play different but organically complementary roles in students’ ability to empathize with one of other.

Our results showed that cognitive empathy contributed to affective empathy between students, hence the students’ skills in recognizing and understanding their peers’ emotions was related to being able to tune to those feelings emotionally. The finding is in line with previous literature suggesting that cognitive dimension is a precedent for affective empathy (see Feshbach and Roe, 1968; Lamm et al., 2010). Accordingly, our results indicate that being able to recognize peers’ affective states is a precondition for being able to sufficiently tune oneself with such experience. Van Lissa et al. (2016) have suggested that of the empathy dimensions, perspective taking is more susceptible to developmental influences in adolescence (Van Lissa et al., 2016). It can be presumed that cognitive empathy, involving perspective taking, is also a central ingredient of co-regulation and prosocial behavior particularly when one does not necessarily agree with the collaborator’s view. However, without affective toning, it is not enough to promote effective co-regulation.

Gendered differences were detected in cognitive and affective empathy. Hence, the results support previous evidence showing that girls typically score higher in empathy (e.g., Jolliffe and Farrington, 2006; Mestre et al., 2009; Calandri et al., 2021). Furthermore, our results showed that gender had an effect on cognitive empathy, and indirect effect on affective empathy implying that, in general, girls scored higher in cognitive empathy, and those girls who had well developed skills in cognitive empathy also scored higher in affective empathy. However, the gender did not have direct effect on affective empathy. This might indicate that gendered differences in students’ empathy stem primarily from differences in their skills in perspective taking and abilities to recognize and understand others’ emotions rather than in their ability to tune into their peers’ emotions.

Further investigation showed that the students’ empathy skills were related to their study wellbeing. We found out that the cognitive empathy contributed negatively to students’ study wellbeing in terms of increased levels of cynicism, inadequacy, and decreased levels of study engagement. Hence, being sensitive to socio-emotional cues, particularly in recognizing peers’ negative emotions, is likely to increase students’ risk of experiencing those burnout symptoms that are more inter-personal in nature, i.e., cynicism and inadequacy, and simultaneously reduce the odds for experiencing study engagement. A reason for the negative association between cognitive empathy and study wellbeing might be that without the affectivity component, cognitive empathy only allows a student to recognize others’ experiences, but not to relate to them, leaving them emotionally deprived of the social fabric of peer relationship. If prolonged, such experience is likely to become painful, potentially further contributing to social exclusion.

In turn, the affective dimension seemed to have a twofold role in promoting the students’ study wellbeing. First, it contributed directly to elevated levels of study wellbeing by reducing the risk of experiencing inadequacy and cynicism and increasing study engagement. The results indicated that students with well-developed affective empathy skills had a lower risk of study burnout and were more likely to feel vigor, dedication, and absorption while studying. A reason for this might be that the students with high levels of affective empathy are more susceptible to getting the contagion of their peers’ positive emotions (see Hatfield et al., 1992; Mendoza and King, 2020), which further increases their study wellbeing. On the other hand, affective empathy may promote study wellbeing by enabling the sharing of engaging study experiences with the peers. Second, affective empathy mediated the association from cognitive empathy to study wellbeing. Accordingly, it seemed that cognitive empathy promoted study wellbeing *via* affective empathy. The results indicate that merely recognizing peers’ emotions and perspective taking can reduce students’ study wellbeing but combined with the ability to tune into their peers’ emotional experiences it can provide a significant resource in promoting students’ study wellbeing.

Contrary to the findings of Farina et al. (2020), we did not detect an association between the empathy dimension and emotional exhaustion. This can partly result from the differences in study designs. It is important to note that empathy skills were measured differently; we focused on empathy between peers, while Farina and others measured empathy in a more general sense. Students’ empathy toward their peers is likely to increase the quality of peer interaction, which can further protect them from emotional exhaustion (see e.g., Ulmanen et al., 2022). However, their general empathy skills might not have direct effects to quality of peer interaction, and therefore, showing empathy to others might more easily lead to depletion of students’ emotional resources. It is also important to keep in mind that most participants in our study were students on the academic track, while participants in the study by Farina et al. (2020) were at a school that prepared students in the helping professions, who have been shown to have high emotional workloads. Another potential explanation for our finding might be that the primary source of exhaustion differs that from cynicism and inadequacy, which has been suggested are more inter-personal in nature, while exhaustion might result from study overload, and hence, is not affected to the same extent by empathy dimensions.

The study provides several directions for future research. First, as the results suggested that the recognition of another’s emotions and perspective taking is not enough, reacting and tuning into other’s emotions are needed to enhance study wellbeing. Accordingly, more studies exploring the teaching practices that enhance both dimensions of empathy are needed. For example, whether positive social support from the teacher could foster students’ abilities to recognize their classmates’ emotions and feel the joy and enthusiasm of another person should be studied. Second, the role of empathy in the spreading of study engagement and study burnout in the classrooms and peer groups should be addressed with longitudinal, nested data sets. Last, the differences in empathy dimensions between students are likely to occur, i.e., some students are likely to score high in both dimensions, some of them in one or the other, and others might have low scores in both dimensions. Knowledge of such empathy profiles could further increase the understanding of the interrelations between empathy dimensions and study wellbeing.

### Practical implications

The findings have implications for promoting student wellbeing, particularly in upper secondary education. It might be that the students with well-developed skills in identifying their peers’ emotions and taking the position of another person, but without affective empathy skills such as tuning into others’ emotions, have a higher risk of study burnout. It could be beneficial to identify these students and monitor and support the quality of their peer interaction intentionally.

When teaching socio-emotional skills, it is important to consider that merely focusing on promoting adolescents’ skills in recognizing and identifying their peers’ emotions is not enough, as it may lead to a decrease in students’ study wellbeing. Alongside promoting students’ skills in looking at things from their peers’ perspective and being able to identify their emotions, the students should be encouraged and trained to react and tune into each other’s feelings, especially to positive ones such as enthusiasm. In other words, these skills should be taught simultaneously.

### Methodological reflections and limitations

Several fit indices (RMSEA, CFI, TLI, and SRMR) indicated that the hypothesized model fit the data. However, according to the Chi-square test, the model fit was not acceptable (Miles and Shevlin, 2007; Iacobucci, 2010).

Cross-sectional design of this study does not allow us to draw causal conclusions. Longitudinal studies are needed to be able to say whether students’ empathy skills contribute to their study wellbeing or another way around. In addition, although the respondents were all over the country, they were not randomly selected students. The girls and those on the academic track were overrepresented in the data, meaning that the sample was not representative of Finnish upper secondary students in these regards. Therefore, the findings cannot be reliably generalized to all upper secondary students in Finland, to other socio-cultural contexts, or other educational contexts. It is also important to keep in mind that the data were collected in the middle of the COVID-19 pandemic, which might have affected both the response rate and the responses, such as the levels of study engagement and burnout.

In this study, we introduced a new instrument designed for measuring students’ empathy toward their peers in educational settings. The instrument included two separate scales: cognitive dimension and affective dimension. Contrary to findings of Baldner and McGinley (2014), the cognitive empathy scale was slightly more internally consistent than the one that assessed affective empathy. However, in our study, based on Cronbach alphas and factor determinacies (Table 1 and Supplementary Appendix 1), the internal consistency of both scales was adequate. However, the reliability and validity of the scales need to be established in other socio-cultural contexts and other age groups.

## Data availability statement

The raw data supporting the conclusions of this article will be made available by the authors, without undue reservation.

## Ethics statement

Ethical review and approval was not required for the study on human participants in accordance with the local legislation and institutional requirements. Written informed consent to participate in this study was provided by the participants’ legal guardian/next of kin.

## Author contributions

LT, HA, KP, TS, and JP contributed to original draft and its editing. LT contributed to conducting the analyses. All authors contributed to the article and approved the submitted version.

## References

[B1] Alloway T. P. Copello E. Loesch M. Soares C. Watkins J. Miller D. (2016). Investigating the reliability and validity of the multidimensional emotional empathy scale. *Meas* 90 438–442. 10.1016/j.measurement.2016.05.014

[B2] Baldner C. McGinley J. (2014). Correlational and exploratory factor analyses (EFA) of commonly used empathy questionnaires: New insights. *Motiv. Emot.* 38 727–744. 10.1007/s11031-014-9417-2

[B3] Bask M. Salmela-Aro K. (2013). Burned out to drop out: Exploring the relationship between school burnout and school dropout. *Eur. J. Psychol. Educ.* 28 511–528. 10.1007/s10212-012-0126-5

[B4] Batanova M. Loukas A. (2011). Social anxiety and aggression in early adolescents: Examining the moderating roles of empathic concern and perspective taking. *J. Youth Adolesc.* 40 1534–1543. 10.1007/s10964-011-9634-x 21293914

[B5] Batanova M. Loukas A. (2012). What are the unique and interacting contributions of school and family factors to early adolescents’ empathic concern and perspective taking? *J. Youth Adolesc* 41 1382–1391. 10.1007/s10964-012-9768-5 22639382

[B6] Boren J. P. (2013). Co-rumination partially mediates the relationship between social support and emotional exhaustion among graduate students. *Commun. Q.* 61 253–267. 10.1080/01463373.2012.751436

[B7] Byrne B. M. (2012). *Structural Equation Modeling with Mplus: Basic Concepts, Applications, and Programming.* London: Routledge, 10.4324/978020380764

[B8] Calandri E. Graziano F. Cattelino E. Testa S. (2021). Depressive symptoms and loneliness in early adolescence: The role of empathy and emotional self-efficacy. *J. Early Adolesc.* 4 369–393. 10.1177/0272431620919156

[B9] Calandri E. Graziano F. Testa S. Cattelino E. Begotti T. (2019). Empathy and depression among early early adolescents: The moderating role of parental support. *Front Psychol* 10:1447. 10.3389/fpsyg.2019.01447 31316426 PMC6610578

[B10] de Wied M. Branje S. J. T. Meeus W. H. J. (2006). Empathy and conflict resolution in friendship relations among adolescents. *Aggr. Behav.* 33 48–55. 10.1002/ab.20166 17441005

[B11] Eisenberg N. (2004). “Empathy and sympathy,” in *Handbook of Emotions*, eds Lewis M. Haviland-Jones J. M. (The Guildford Press), 677–691.

[B12] Eisenberg N. Shea C. L. Carlo G. Knight G. P. (1991). “Empathy-related responding and cognition: A “chicken and egg” dilemma,” in *Handbook of Moral Behavior and Development*, eds Kurtinez W. M. Gewirtz J. L. 63–88.

[B13] Euler F. Steinlin C. Stadler C. (2017). Distinct profiles of reactive and proactive aggression in adolescents: Associations with cognitive and affective empathy. *Child Adolesc. Psychiatry Ment. Health.* 11:1. 10.1186/s13034-016-0141-4 28077965 PMC5217447

[B14] Farina E. Ornaghi V. Pepe A. Fiorilli C. Grazzarini I. (2020). High school student burnout: Is empathy a protective or risk factor? *Front. Psychol.* 13:897. 10.3389/fpsyg.2020.00897 32477218 PMC7237742

[B15] Feshbach N. D. Roe K. (1968). Empathy in six- and seven-year-olds. *Child Dev.* 39 133–145. 10.2307/1127365 5645790

[B16] Finnish Institute for Health and Welfare (2021). *School Health Promotion Study.* Available online at: https://thl.fi/fi/web/lapset-nuoret-ja-perheet/tutkimus-ja-seuranta/varhaiskasvatus-koulunkaynti-ja-opiskelu (accessed June 17, 2022).

[B17] Finnish National Board on Research Integrity (2019). *The Ethical Principles Of Research With Human Participants And Ethical Review In The Human Sciences In Finland.* Available online at https://tenk.fi/sites/default/files/2021-66801/Ethical_review_in_human_sciences_2020.pdf (accessed February 22, 2022).

[B18] Fornell C. Larcker D. F. (1981). Evaluating structural equation models with unobservable variables and measurement error. *J. Mark. Res.* 18 39–50. 10.2307/3151312

[B19] Hair J. F. Black W. C. Babin B. J. Anderson R. E. (2014). *Multivariate Data Analysis*, 7th Edn. Harlow: Pearson.

[B20] Hatfield E. Cacioppo J. T. Rapson R. L. (1992). “Primitive emotional contagion,” in *Emotion and Social Behavior*, ed. Clark M. S. (Sage Publications), 151–177.

[B21] Iacobucci D. (2010). Structural equations modeling: Fit indices, sample size, and advanced topics. *J. Consum. Psychol.* 20 90–98. 10.1016/j.jcps.2009.09.003

[B22] Jolliffe D. Farrington D. P. (2006). Development and validation of the basic empathy scale. *J. Adolesc.* 29 589–611. 10.1016/j.adolescence.2005.08.010 16198409

[B23] Kiuru N. Aunola K. Nurmi J. E. Leskinen E. Salmela-Aro K. (2008). Peer group influence and selection in adolescents’ school burnout. *Merrill Palmer Q.* 54 23–55. 10.1353/mpq.2008.0008 34409987

[B24] Kock N. Lynn G. S. (2012). Lateral collinearity and misleading results in variance-based sem: An illustration and recommendations. *J. Assoc. Inf. Syst.* 13 546–580. 10.17705/1jais.00302

[B25] Korhonen J. Linnanmäki K. Aunio P. (2014). Learning difficulties, academic wellbeing and educational dropout: A person-centred approach. *Learn. Individ. Differ.* 31 1–10. 10.1016/j.lindif.2013.12.011

[B26] Laghi F. Lonigro A. Pallini S. Baiocco R. (2018). Emotion regulation and empathy: Which relation with social conduct? *J. Genet. Psychol.* 179 62–70. 10.1080/00221325.2018.1424705 29384468

[B27] Lamm C. Meltzoff A. N. Decety J. (2010). How do we empathize with someone who is not like us? A functional magnetic resonance imaging study. *J. Cogn. Neurosci.* 22 362–376. 19199417 10.1162/jocn.2009.21186

[B28] Lewis A. D. Huebner E. S. Malone P. S. Valois R. F. (2011). Life satisfaction and student engagement in adolescents. *J. Youth Adolesc.* 40 249–262. 10.1007/s10964-010-9517-6 20204687

[B29] MacArthur K. R. Stacey C. L. Harvey S. Markle J. (2021). The direct and indirect effects of clinical empathy on wellbeing among pre-medical students: A structural equation model approach. *BMC Med. Educ.* 21:412. 10.1186/s12909-021-02838-x 34340661 PMC8327048

[B30] Mehrabian A. Epstein N. (1972). A measure of emotional empathy. *J. Pers.* 40 525–543. 10.1111/j.1467-6494.1972.tb00078.x 4642390

[B31] Mendoza N. B. King R. B. (2020). The social contagion of student engagement in school. *J. Pers Soc. Psychol.* 41 454–474. 10.1177/0143034320946803

[B32] Mestre M. Samper P. Frías M. Tur A. (2009). Are women more empathetic than men? A longitudinal study in adolescence. *Span J. Psychol.* 12 76–83. 10.1017/S1138741600001499 19476221

[B33] Miles J. Shevlin M. (2007). A time and a place for incremental fit indices. *Pers Individ. Differ.* 42 869–874. 10.1016/j.paid.2006.09.022

[B34] Muthén Muthén B. O. (1998–2017). *Mplus User’s Guide*, 8th Edn. Los Angeles, CA: Muthén & Muthén.

[B35] Raine A. Chen F. R. (2018). The cognitive, affective, and somatic empathy scales (CASES) for children. *J. Clin. Child Adolesc. Psychol.* 47 24–37. 10.1080/15374416.2017.1295383 28318335

[B36] Rubin K. H. Bukowski W. Parker J. Bowker J. C. (2008). “Peer interactions, relationships, and groups,” in *Developmental Psychology: An Advanced Course*, eds Damon W. Lerner R. (New York: Wiley).

[B37] Ryan A. M. (2001). The peer group as a context for the development of young adolescent motivation and achievement. *Child Dev.* 72 1135–1150. 11480938 10.1111/1467-8624.00338

[B38] Salmela-Aro K. Tuominen-Soini H. (2010). Adolescents’ life satisfaction during the transition to post-comprehensive education: Antecedents and consequences. *J. Happiness Stud.* 11 683–701. 10.1007/s10902-009-9156-3

[B39] Salmela-Aro K. Upadyaya K. (2012). The schoolwork engagement inventory. *Eur. J. Psychol. Assess.* 28 60–67. 10.1027/1015-5759/a000091

[B40] Salmela-Aro K. Upadyaya K. (2014). School burnout and engagement in the context of demands–resources model. *Br. J. Educ. Psychol.* 84 137–151. 10.1111/bjep.12018 24547758

[B41] Salmela-Aro K. Upadyaya K. (2020). School engagement and school burnout profiles during high school – The role of socio-emotional skills. *Eur. J. Dev. Psychol*. 17, 943–964. 10.1080/17405629.2020.1785860

[B42] Salmela-Aro K. Kiuru N. Leskinen E. Nurmi J.-E. (2009a). School burnout inventory: Reliability and validity. *Eur. J. Psychol. Assess.* 25 48–57. 10.1027/1015-5759.25.1.48

[B43] Salmela-Aro K. Savolainen H. Holopainen L. (2009b). Depressive symptoms and school burnout during adolescence: Evidence from two cross-lagged longitudinal studies. *J. Youth Adolesc.* 38 1316–1327. 19779808 10.1007/s10964-008-9334-3

[B44] Salmela-Aro K. Upadyaya K. Vinni-Laakso J. Hietajärvi L. (2021). Adolescents’ longitudinal school engagement and burnout before and during COVID-19—the role of socio-emotional skills. *J. Res. Adolesc.* 31 796–807. 10.1111/jora.12654 34448301 PMC8646577

[B45] Schafer J. L. Graham J. W. (2002). Missing data: Our view of the state of the art. *Psychol. Methods.* 7 147–177. 10.1037/1082-989X.7.2.147 12090408

[B46] Schaufeli W. Salanova M. Gonzalez-Roma V. Bakker A. (2002). The Measurement of engagement and burnout: A two sample confirmatory factor analytic approach. *J. Happ. Stud.* 3 71–92. 10.1023/A:1015630930326

[B47] Schwartz-Mette R. A. Rose A. J. (2012). Co-rumination mediates contagion of internalizing symptoms within youths’ friendships. *Dev. Psychol.* 48 1355–1365. 10.1037/a0027484 22369336 PMC3371303

[B48] Statistics Finland (2020). *Suomen Virallinen Tilasto (SVT): Koulutukseen Hakeutuminen.* Available online at: http://www.stat.fi/til/khak/2020/khak_2020_2021-12-09_tie_001_fi.html (accessed: September 6, 2022)

[B49] Symonds J. Schoon I. Salmela-Aro K. (2016). Developmental trajectories of emotional disengagement from schoolwork and their longitudinal associations in England. *Br. Educ. Res. J.* 42 993–1022.

[B50] Ulmanen S. Soini T. Pietarinen J. Pyhältö K. Rautanen P. (2022). Primary and lower secondary school students’ social support profiles and study wellbeing. *J. Early Adolesc.* 42 613–646. 10.1177/02724316211058061

[B51] Usán Supervía P. Salavera Bordás C. Teruel Melero P. (2019). Approaching the relationship between emotional intelligence, burnout and academic commitment in students of compulsory secondary education. *Electron. J. Res. Educ. Psychol.* 17 541–560.

[B52] van der Graaff J. Branje S. De Wied M. Hawk S. Van Lier P. Meeus W. (2018). Perspective taking and empathic concern in adolescence: Gender differences in developmental changes. *Dev. Psychol.* 50:881. 10.1037/a0034325 24040846

[B53] Van Lissa C. J. Hawk S. T. Branje S. Koot H. M. Meeus W. H. J. (2016). Common and unique associations of adolescents’ affective and cognitive empathy development with conflict behavior towards parents. *J. Adolesc.* 47 60–70. 10.1016/j.adolescence.2015.12.005 26760479

[B54] Vinayak S. Judge J. (2018). Resilience and empathy as predictors of psychological wellbeing among adolescents. *Int. J. Health Sci.* 8 192–200.

[B55] Vinter K. Aus K. Arro G. (2021). Adolescent girls’ and boys’ academic burnout and its associations with cognitive emotion regulation strategies. *Educ. Psychol.* 41 1061–1077. 10.1080/01443410.2020.1855631

[B56] Wagaman M. A. Geiger J. M. Shockley C. Segal E. A. (2015). The role of empathy in burnout, compassion satisfaction, and secondary traumatic stress among social workers. *Soc. Work.* 60 201–209. 10.1093/sw/swv014 26173361

[B57] Walburg V. (2014). Burnout among high school students: A literature review. *Child Youth Serv. Rev.* 42 28–33. 10.1016/j.childyouth.2014.03.020

[B58] Widlund A. Tuominen H. Korhonen J. (2018). Academic wellbeing, mathematics performance, and educational aspirations in lower secondary education: Changes within a school year. *Front. Psychol.* 9:297. 10.3389/fpsyg.2018.00297 29593603 PMC5859340

[B59] Wing M. N. LaRusso M. D. Smith R. L. (2021). Teacher empathy and students with problem behaviors: Examining teachers’ perceptions, responses, relationships, and burnout. *Psychol. Sch.* 58 1575–1596. 10.1002/pits.22516

[B60] Yang H. Chen J. (2016). Learning perfectionism and learning burnout in a primary school student sample: A test of a learning-stress mediation model. *J. Child. Fam. Stud.* 25 345–353. 10.1007/s10826-015-0213-8

