# Supplementary material for: The role of empathy between peers in upper secondary students’ study engagement and burnout
Source: Front Psychol. 2022 Sep 30;13:978546. doi: 10.3389/fpsyg.2022.978546 (PMC9561899; doi:10.3389/fpsyg.2022.978546)
Supplement: Supplementary file 1 [file Table_1.docx]

**Appendix 1.** The scales, items, standardized factor loadings, item reliability coefficients (R^2^), factor determinacies, AVE and CR values for the scales (items translated from Finnish).

| **Scales and items** | Standardized factor loadings | *R*^2^ | Factor determinacy | AVE | CR |
| --- | --- | --- | --- | --- | --- |
| **Cognitive empathy** |  |  | .96 | .64 | .90 |
| CE01 I can tell if my peer is upset, even if they don’t tell me | .771 | .594 |  |  |  |
| CE02 It is easy for me to put myself in my peer’s shoes if they tell me they are disappointed. | .755 | .570 |  |  |  |
| CE03 I recognize the feelings expressed by my peers. | .923 | .852 |  |  |  |
| CE04 If my peers are in a good mood, I recognize it from their facial expressions or their behavior. | .837 | .700 |  |  |  |
| CE05 I can easily understand how someone feels in different situations. | .702 | .493 |  |  |  |
| **Affective empathy** |  |  | .92 | .60 | .86 |
| AE01 It is easy for me to be happy for my peers. | .725 | .526 |  |  |  |
| AE02 I am worried if my peers have concerns. | .857 | .735 |  |  |  |
| AE03 I react easily to the enthusiasm of my peers. | .791 | .625 |  |  |  |
| AE04 I get excited easily if my peers are excited. | .726 | .527 |  |  |  |
| **Exhaustion** |  |  | .94 | .62 | .83 |
| EXH01 I feel drowned by my school work. | .728 | .531 |  |  |  |
| EXH02 I often sleep poorly due to issues with my school work. | .773 | .598 |  |  |  |
| EXH03 I spend a lot of time worrying about my studies outside of school time. | .852 | .726 |  |  |  |
| **Cynicism** |  |  | .97 | .86 | .92 |
| CYN01 I feel like my studies are no longer important. | .967 | .935 |  |  |  |
| CYN02 Going to school feels like a waste of time to me. | .884 | .781 |  |  |  |
| **Inadequacy** |  |  | .95 | .85 | .74 |
| INAD01 I feel inadequate in relation to my studies. | .868 | .753 |  |  |  |
| INAD02 I often feel that I am failing in my studies. | .848 | .719 |  |  |  |
| **Study engagement** |  |  | .97 | .67 | .95 |
| ENG01 When I study, I feel like I am bursting with energy. | .870 | .757 |  |  |  |
| ENG02 I find my studies to be full of meaning and purpose. | .757 | .573 |  |  |  |
| ENG03 Time flies when I’m studying. | .764 | .584 |  |  |  |
| ENG04 When studying, I feel strong and vigorous. | .840 | .705 |  |  |  |
| ENG05 I am enthusiastic about my studies. | .915 | .838 |  |  |  |
| ENG06 When I am studying, I forget everything else around me. | .780 | .608 |  |  |  |
| ENG07 When I get up in the morning, I feel like going to class. | .824 | .680 |  |  |  |
| ENG08 My studies inspire me. | .862 | .742 |  |  |  |
| ENG09 I can get carried away by my studies. | .750 | .562 |  |  |  |
